# Supplementary figures and images for: Blocking HXA3-mediated neutrophil elastase release during S. pneumoniae lung infection limits pulmonary epithelial barrier disruption and bacteremia
Source: mBio. 2024 Aug 9;15(9):e01856-24. doi: 10.1128/mbio.01856-24 (PMC11389395; doi:10.1128/mbio.01856-24)

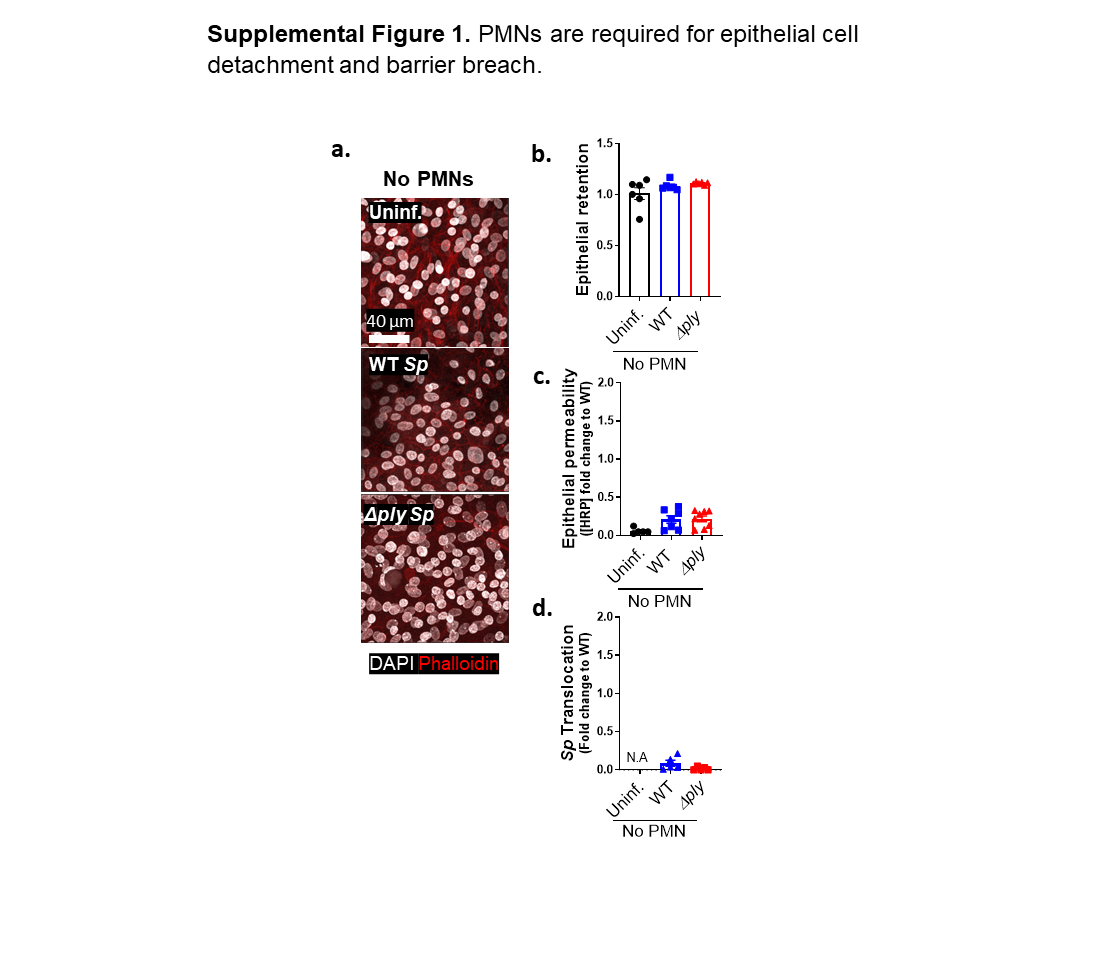

Supplement: Figure S1 — PMNs are required for epithelial cell detachment and barrier breach. [file mbio.01856-24-s0001.tiff]

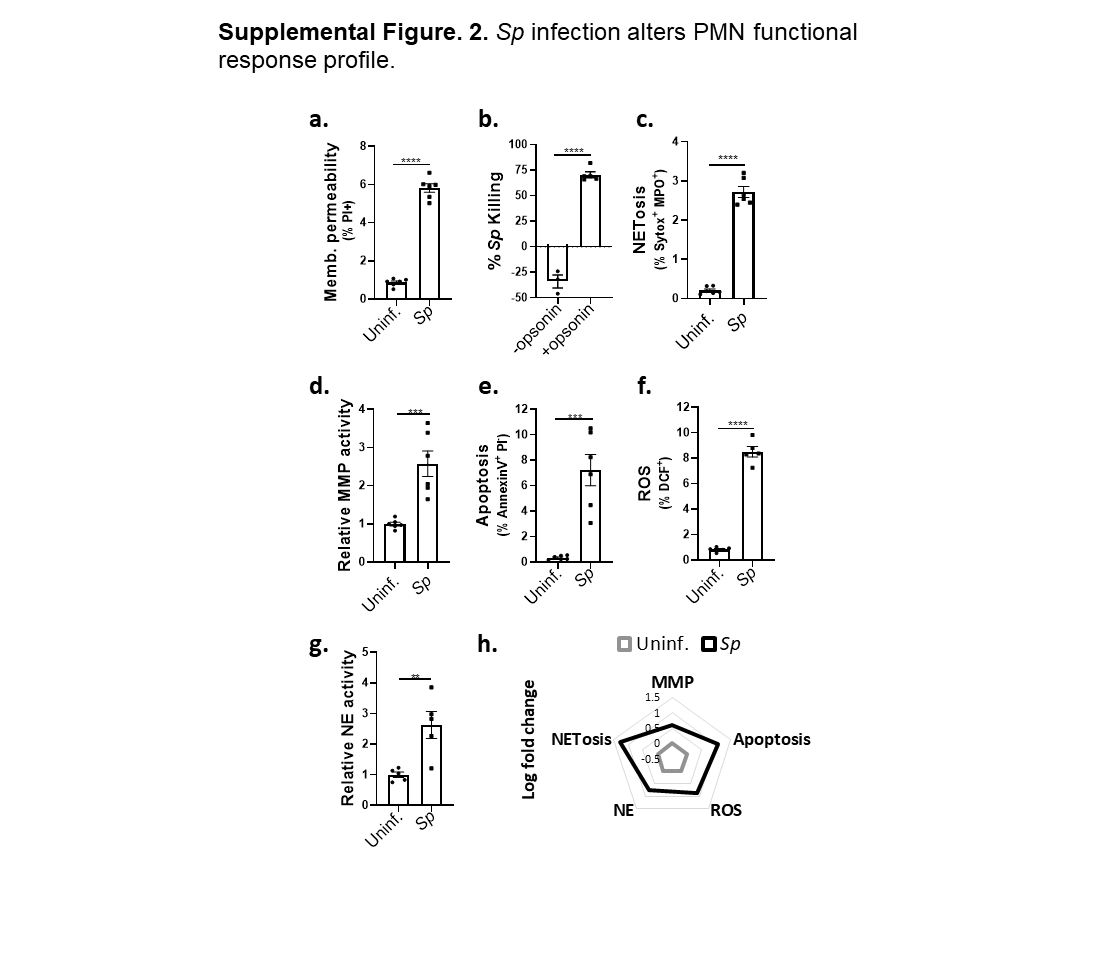

Supplement: Figure S2 — Sp infection alters PMN functional response profile. [file mbio.01856-24-s0002.tiff]
