# Supplementary material for: Blocking HXA3-mediated neutrophil elastase release during S. pneumoniae lung infection limits pulmonary epithelial barrier disruption and bacteremia
Source: mBio. 2024 Aug 9;15(9):e01856-24. doi: 10.1128/mbio.01856-24 (PMC11389395; doi:10.1128/mbio.01856-24)
Supplement: Legends — for Fig. S1 and S2. [file mbio.01856-24-s0003.pdf]

Supplemental Figure Legends

Supplemental Figure 1. PMNs are required for epithelial cell detachment and barrier breach. Human BSC-derived ALI monolayers were apically infected with 1 × 10^7 WT or *Δply Sp* without basolateral PMNs. **(a)** After two hours, monolayer integrity was assessed by fluorescence confocal microscopy after staining nuclei with DAPI and F-actin with fluorescent phalloidin. For clarity, images shown are of extended projections (all z-sections collapsed into 1 plane). Scale bar = 40 µm for all images. **(b)** Epithelial retention was quantitated by enumerating epithelial cell nuclei relative to uninfected ALI. **(c)** Epithelial permeability was measured by HRP flux relative to monolayers infected with WT *Sp*. **(d)** *Sp* translocation was quantitated by measuring basolateral CFU. Each panel is a representative of three independent experiments. Error bars represent mean ± SEM.

Supplemental Figure 2. *Sp* infection alters PMN functional response profile. 1 × 10^6 PMNs were uninfected or infected with 1 × 10^7 *Sp* and evaluated for functional performance via **(a)** PMN membrane permeability determined by propidium iodide staining (PI^+), **(b)** opsonophagocytic killing with or without the addition of complement opsonin, quantitated by plating for CFU, **(c)** NETosis determined by Sytox and anti-MPO staining ($Sytox^+$ MPO^+), **(d)** released MMP activity by substrate conversion and expressed relative to uninfected PMNs, **(e)** apoptosis determined by lack of straining by propidium iodide and positive staining of Annexin V (PI^- Annexin V^+), **(f)** ROS production by intracellular oxidation of substrate (DCF^+), or **(g)** released NE activity by substrate conversion and expressed relative to uninfected PMNs (See Materials and Methods). **(h)** Radar plot summary of log fold change in PMN functional performance in **(c-g)**. Each panel shown is representative of three independent experiments. Error bars represent mean ± SEM. Statistical analysis was performed using ordinary one-way ANOVA: **p-value < 0.01, ***p-value < 0.001, ****p-value < 0.0001.
